# The Impact of a Line Probe Assay Based Diagnostic Algorithm on Time to Treatment Initiation and Treatment Outcomes for Multidrug Resistant TB Patients in Arkhangelsk Region, Russia

**DOI:** 10.1371/journal.pone.0152761

**Published:** 2016-04-07

**Authors:** Platon Eliseev, Grigory Balantcev, Elena Nikishova, Anastasia Gaida, Elena Bogdanova, Donald Enarson, Tara Ornstein, Anne Detjen, Russell Dacombe, Elena Gospodarevskaya, Patrick P. J. Phillips, Gillian Mann, Stephen Bertel Squire, Andrei Mariandyshev

**Affiliations:** 1 Northern State Medical University, Arkhangelsk, Russian Federation; 2 Northern Arctic Federal University, Arkhangelsk, Russian Federation; 3 Arkhangelsk Clinical Antituberculosis Dispensary, Arkhangelsk, Russian Federation; 4 The International Union Against Tuberculosis and Lung Disease, Paris, France; 5 Liverpool School of Tropical Medicine, Liverpool, United Kingdom; 6 MRC Clinical Trials Unit at UCL, London, United Kingdom; Hospital San Agustín. Aviles. Asturias. Spain, SPAIN

## Abstract

**Background:**

In the Arkhangelsk region of Northern Russia, multidrug-resistant (MDR) tuberculosis (TB) rates in new cases are amongst the highest in the world. In 2014, MDR-TB rates reached 31.7% among new cases and 56.9% among retreatment cases. The development of new diagnostic tools allows for faster detection of both TB and MDR-TB and should lead to reduced transmission by earlier initiation of anti-TB therapy.

**Study Aim:**

The PROVE-IT (Policy Relevant Outcomes from Validating Evidence on Impact) Russia study aimed to assess the impact of the implementation of line probe assay (LPA) as part of an LPA-based diagnostic algorithm for patients with presumptive MDR-TB focusing on time to treatment initiation with time from first-care seeking visit to the initiation of MDR-TB treatment rather than diagnostic accuracy as the primary outcome, and to assess treatment outcomes. We hypothesized that the implementation of LPA would result in faster time to treatment initiation and better treatment outcomes.

**Methods:**

A culture-based diagnostic algorithm used prior to LPA implementation was compared to an LPA-based algorithm that replaced BacTAlert and Löwenstein Jensen (LJ) for drug sensitivity testing. A total of 295 MDR-TB patients were included in the study, 163 diagnosed with the culture-based algorithm, 132 with the LPA-based algorithm.

**Results:**

Among smear positive patients, the implementation of the LPA-based algorithm was associated with a median decrease in time to MDR-TB treatment initiation of 50 and 66 days compared to the culture-based algorithm (BacTAlert and LJ respectively, p<0.001). In smear negative patients, the LPA-based algorithm was associated with a median decrease in time to MDR-TB treatment initiation of 78 days when compared to the culture-based algorithm (LJ, p<0.001). However, several weeks were still needed for treatment initiation in LPA-based algorithm, 24 days in smear positive, and 62 days in smear negative patients. Overall treatment outcomes were better in LPA-based algorithm compared to culture-based algorithm (p = 0.003). Treatment success rates at 20 months of treatment were higher in patients diagnosed with the LPA-based algorithm (65.2%) as compared to those diagnosed with the culture-based algorithm (44.8%). Mortality was also lower in the LPA-based algorithm group (7.6%) compared to the culture-based algorithm group (15.9%). There was no statistically significant difference in smear and culture conversion rates between the two algorithms.

**Conclusion:**

The results of the study suggest that the introduction of LPA leads to faster time to MDR diagnosis and earlier treatment initiation as well as better treatment outcomes for patients with MDR-TB. These findings also highlight the need for further improvements within the health system to reduce both patient and diagnostic delays to truly optimize the impact of new, rapid diagnostics.

## Background

Multidrug-resistant (MDR) tuberculosis (TB) is one of the main global public health challenges facing mankind in the XXI century. According to the latest World Health Organization (WHO) report [1], an estimated 480 000 people developed MDR-TB worldwide in 2014, and there were an estimated 190 000 deaths from MDR-TB. Data from drug resistance surveys and continuous surveillance among notified TB cases suggest that 3.5% of newly diagnosed TB cases, and 21% of those previously treated for TB had MDR-TB [2]. In the Arkhangelsk region of northern Russia, MDR-TB rates among new cases are amongst the highest in the world [3]. Following the implementation of DOTS (directly observed treatment, short-course), the TB incidence in this region declined from 118.7/100 000 in 2001 to 39.7/100 000 in 2015. Mortality declined from 15.2 to 4.7/100 000 over the same period [4]. Despite the decline in total number of TB cases, MDR-TB rates among patients remain high. In 2014, MDR-TB was detected in 31.7% of new cases and in 56.9% among retreatment cases. That same year, 115 patients with a first episode of MDR-TB were registered in the region.

The development of new diagnostic tools allows for faster detection of both TB and MDR-TB and could lead to reduced transmission by earlier initiation of anti TB therapy [5]. In 2008, the WHO recommended the Line Probe Assay (LPA) as a rapid diagnostic tool to define drug susceptibility of *Mycobacterium tuberculosis* (*M*.*tb*) in smear positive specimens or in isolates of *M*.*tb* grown from smear negative specimens [6]. This recommendation was made based on the accuracy of LPA for diagnosing both TB and MDR-TB [7]. However, there are few data on the test’s impact on general epidemiological outcomes such as incidence, prevalence, mortality, treatment outcomes, as well as on the practical challenges and costs related to its implementation for both patients and the health system.

In 2009, under the USAID-funded (United States Agency for International Development) TREAT TB initiative, Northern State Medical University (NSMU) in collaboration with The International Union Against Tuberculosis and Lung Disease (The Union) and partners in South Africa, Brazil and at the Liverpool School of Tropical Medicine (LSTM) developed the PROVE-IT studies (Policy Relevant Outcomes from Validating Evidence on Impact). These studies were built around an impact assessment framework (IAF), that includes 5 layers of analysis (effectiveness analysis, equity analysis, health system, scale up analysis and policy analysis), and aim to comprehensively assess new TB diagnostic tests within the health system context in different epidemiological settings and to define the measures needed to successfully implement new diagnostics within health systems [8].

PROVE-IT Russia aimed to assess the influence of LPA as part of the diagnostic algorithm for patients with presumptive MDR-TB on the time from first-care seeking visit to the initiation of MDR-TB treatment as well as patient outcomes after 20 months of treatment.

## Methods

### Setting

The study was conducted in Arkhangelsk, northwest Russia. With a population of 1.12 million, Arkhangelsk lies in a region with a circumpolar surface area of 410 000 square kilometers. There are 20 districts in the region with more than 50 hospitals and outpatient clinics [9].

The specialized TB control services in the Arkhangelsk region consist of a regional dispensary, district ambulatory TB units, as well as a tuberculosis hospital and a TB colony in the penitentiary system according to TB control policies in the Russian Federation [10]. The Arkhangelsk Clinical Anti-tuberculosis Dispensary (ACAD) is a central facility performing diagnosis and treatment of TB in the region. All patients diagnosed with TB in the region are routinely tested for HIV. Due to the high rates of MDR-TB, all presumed TB cases are tested for drug susceptibility (DST) at ACAD. LPA for both first and second line DST was routinely implemented at ACAD in 2009 (Hain Lifescience Genotype MTBDR*plus* and MTBDR*sl*).

### Study population

**Fig 1:**
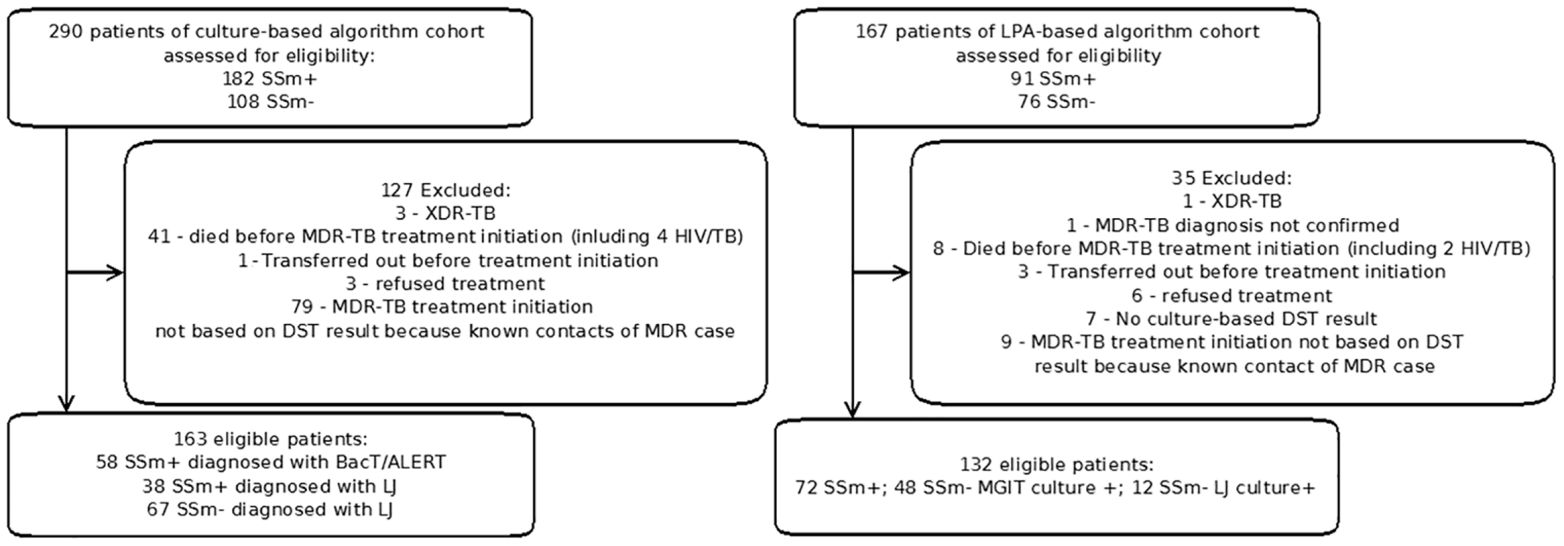
Study population. 290 MDR-TB patients were registered from September 2007 to August 2009 for the culture-based algorithm. 163 MDR-TB patients were included in the study and 127 were excluded. 167 MDR-TB cases were registered from April 2011 to July 2012 for LPA-based algorithm. 132 MDR-TB patients were included in the study and 35 were excluded.

We included all patients managed in the civil population with a first episode of MDR-TB. The following patients were excluded from the analysis: contacts of a MDR case who were presumptively treated with a MDR-TB regimen but lacked a DST result, MDR-TB patients who were found to have primary extensively drug-resistant (XDR) TB on testing for second-line drug sensitivities before MDR-TB treatment was initiated, died before MDR-TB treatment was initiated, transferred out before treatment initiation, refused treatment during the first 2 weeks after diagnosis, and MDR-TB diagnosis not confirmed by phenotypic method (or no culture-based DST results) (Fig 1).

### Study design

**Fig 2:**
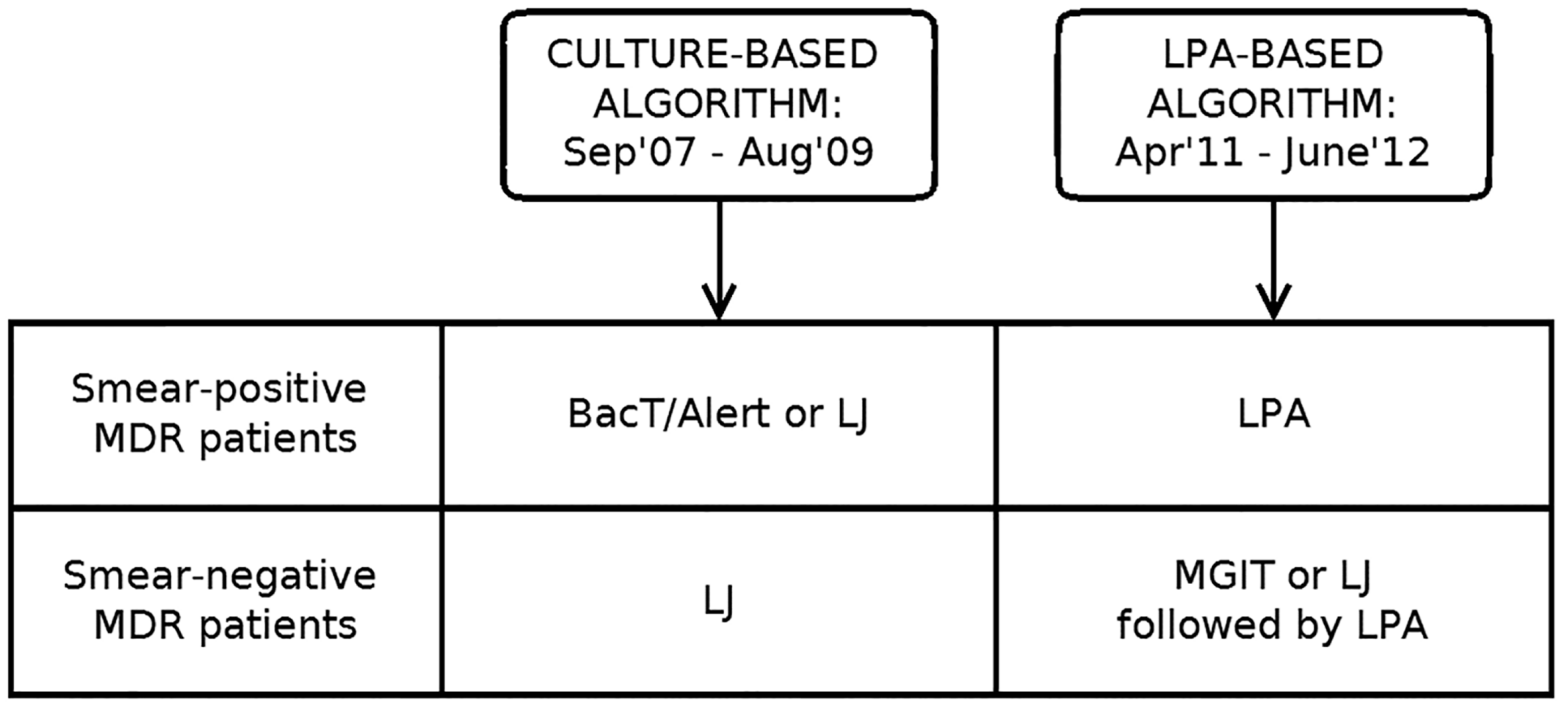
Study design, comparison of culture-based and LPA-based diagnostic algorithms for MDR-TB used at ACAD between 2007 and 2012. Two diagnostic algorithms among MDR-TB patients were compared: culture-based algorithm (data collected from September 2007 to August 2009) and LPA-based algorithm used from September 2009 after LPA implementation (data collected from April 2011 to June 2012).

To assess the primary outcome, ‘time from first care-seeking visit to MDR treatment initiation’, we compared two diagnostic algorithms among MDR-TB patients: the culture-based algorithm before LPA implementation to a LPA-based algorithm used from September 2009 after LPA implementation (Fig 2). Data culture-based algorithm were collected from September 2007 to August 2009 and LPA-based algorithm from April 2011 to June 2012. Patients were not enrolled in the study for prospective LPA-based algorithm until ethical permission from ethics committee of Northern State Medical University in Arkhangelsk, Russian Federation and by Ethics Advisory Group at The International Union Against Tuberculosis and Lung Disease in 2011.

MDR-TB patients who were diagnosed with the culture-based algorithm were enrolled retrospectively. For those patients, culture and DST were done by either BacTAlert in smear positive (SSm+) or by Löwenstein Jensen (LJ) in smear negative (SSm-) and a subgroup of SSm+ in whom BacTAlert result was unavailable due to no growth or contamination.

Under the LPA-based algorithm we prospectively included patients where LPA, as a main DST test, was implemented, replacing BacTAlert and LJ. LPA was performed directly on specimens from SSm+ patients, and on isolates obtained by MGIT culture on specimens from SSm- patients. In cases where MGIT culture was unavailable due to no growth or contamination, LPA was performed on isolates obtained from LJ culture, which had been done in parallel. Per national guidelines, patients included in this analysis were not initiated on treatment for MDR in both culture-based and LPA-based algorithms until DST results were available. For each algorithm, there were slightly different approaches for SSm+ and SSm- patients. SSm+ patients are usually admitted to the in-patient department at ACAD. SSm- patients are managed at the district ambulatory TB units, but specimens are sent to ACAD for culture and DST. All cultures and DST are performed according to national and manufacturer’s recommendations [11–14].

BacTAlert was the main DST method for detecting MDR-TB in the region before LPA was introduced but due to limited resources and relatively high price of the reagents it was performed only for SSm+ group of patients as they were considered the main source of TB transmission. Data were collected for all eligible patients in both algorithms over a period of approximately 18 months.

Various time points along the diagnostic path way of each algorithm were extracted from the ACAD electronic database and medical records in order to assess our primary outcome ‘time from first-care seeking visit to treatment initiation’. Different time components of the diagnostic pathway used in each algorithm were included to measure time to treatment initiation: time from first care seeking visit to first microscopy, time from first microscopy to submission for culture and conventional DST result or LPA, laboratory turn-around time for LPA and conventional DST, time from laboratory MDR-TB confirmation to treatment initiation

To assess additional secondary outcome measures, we collected data on: smear conversion rate at 2 months; LJ culture conversion rates at 2 and 6 months; treatment success rate, lost to follow up rate; all-cause mortality at any point; and the proportion of cases that developed XDR-TB. All patients were followed for 20 months and treatment outcomes recorded.

MDR-TB was defined as resistance to isoniazid and rifampicin, with or without resistance to other first-line drugs. XDR-TB was defined as resistance to at least isoniazid and rifampicin, and to any fluoroquinolone, and to any of the three second-line injectables (amikacin, capreomycin, and kanamycin). Treatment outcomes were defined according to WHO recommendations [15]. MDR-TB patients received the same type of treatment in both algorithms according to WHO recommendation.

### Analysis and Statistical Methods

All data were obtained from official medical documents and an electronic recording and reporting system called INIT-TB, which has been used in ACAD for routine registration of TB cases, test results and treatment outcomes since 2007. Double data entry was used for all information. Statistical analyses were performed using Microsoft Excel 2010, Mathworks MATLAB 2009.

For each sub-group of patients, the different components defining time to treatment initiation were analyzed, and median values calculated. Treatment outcomes were compared using Fisher’s exact test on the 6-by-2 table to evaluate whether the distribution of treatment outcomes differed between culture-based and LPA-based algorithms. Mann-Whitney U-test was used for comparison between cohorts diagnosed with the culture-based versus LPA-based algorithm.

### Sample Size

The sample sizes were calculated for the primary analysis of a t-test on log-transformed times comparing the culture-based and the LPA-based algorithm using standard formulae. The standard deviation (sd) of the log-transformed times in each group was assumed to be 0.3, based on data from a similar study in South Africa [16].

No data were available before the study on reduction in time to MDR-TB treatment associated with implementation of LPA, but from our personal experience we expected a reduction of at least 15 days in SSm+ and at least 7 days in SSm- groups (from 30 and 37 days respectively). For SSm+ patients, we calculated that we would need 24 confirmed MDR-TB patients to demonstrate a reduction under the LPA-based algorithm to 15 days with 90% power. For SSm- patients, 190 confirmed MDR patients would be required with 80% power.

The study was approved by the ethics committee of Northern State Medical University in Arkhangelsk, Russian Federation (approval protocol № 07/06) and by Ethics Advisory Group at The International Union Against Tuberculosis and Lung Disease (approval protocol № 01/11). Statistical data from Ministry of Health was used for the study for historical cohort. A waiver of informed consent was granted for the use of routine data. Additionally informed consent was given by participants for their clinical records to be used in this study for current cohort after 2011. All patient records information was anonymized and de-identified prior to analysis.

## Results

### Impact of the introduction of LPA on time to treatment initiation

During the assessment period for the culture-based algorithm from September 2007 to August 2009, 1203 TB cases (1042 new cases and 161 retreatment cases) were registered, among them 14 had HIV/TB coinfection– 8 susceptible TB or without bacteriological confirmation and 6 MDR-TB. MDR-TB was isolated from 290 of these patients and we included 163 in the study, 96 SSm+ and 67 SSm-. The reasons for excluding 127 patients are given in Fig 1. For assessment of the LPA-based algorithm from April 2011 to July 2012, 876 TB cases were registered (718 new cases and 158 retreatment cases), among them 9 had HIV/TB coinfection– 7 susceptible TB or without bacteriological confirmation and 2 MDR-TB. MDR-TB was isolated from 167 of these patients and we included 132 patients in the study, 72 SSm+ and 60 SSm-. The reasons for excluding 35 patients are given in Fig 1. The main characteristics of the 295 included patients are shown in Table 1.

**Table 1:** Data MDR-TB patients in culture-based and LPA-based algorithm.

	Total	Culture-based algorithm	LPA-based algorithm
N	295	163	132
smear+ n (%)	168 (56.9%)	96 (58.9%)	72 (54.5%)
smear—n (%)	127 (43.1%)	67 (41.1%)	60 (45.5%)
Male (%)	78.3	80.9	75.0
HIV-infected	2	2	0
Average age, years (Median, SD)	41.9±12.2 IQR 32–51	42.1±11.5 IQR 33–50.5	41.6±12.9 IQR 31–51

**Fig 3:**
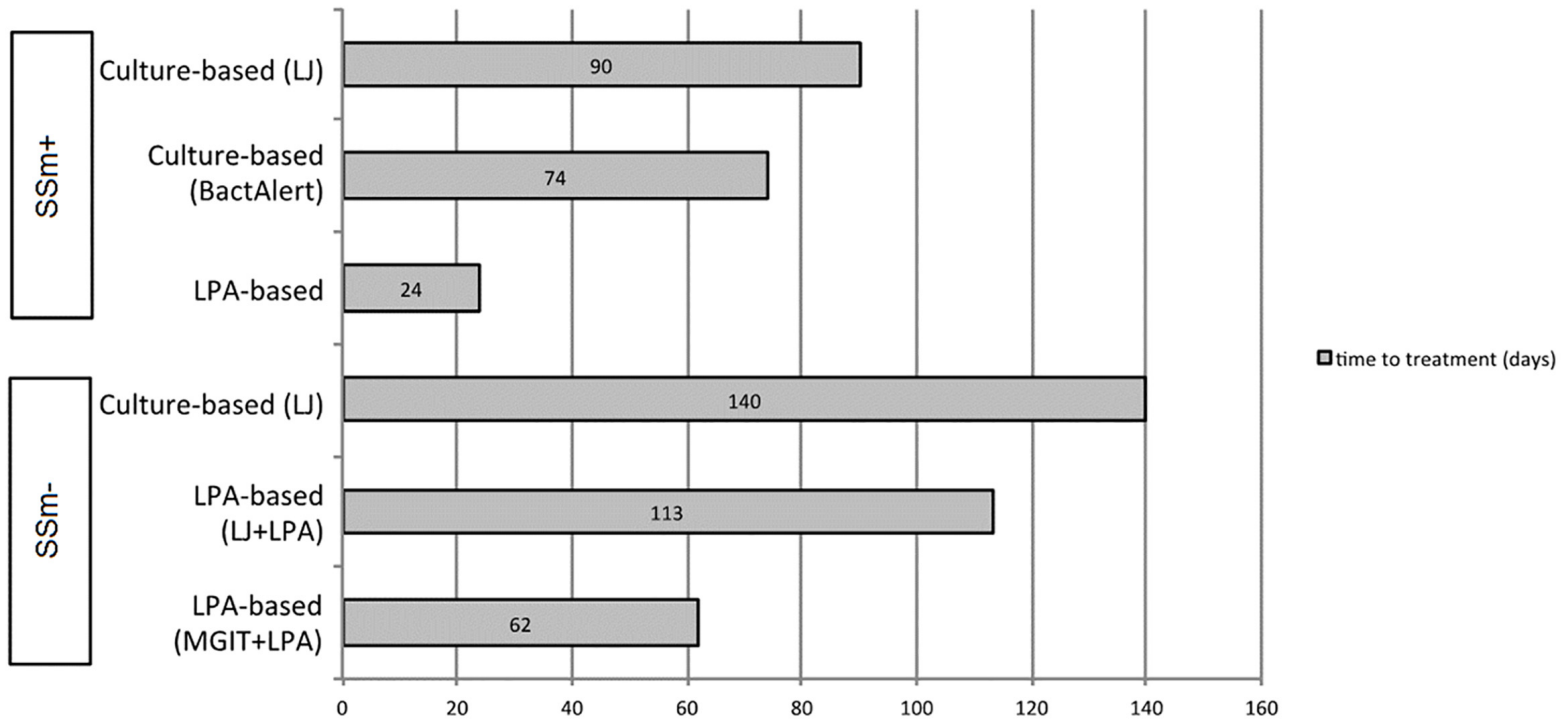
Median time to treatment initiation from first-care seeking visit for SSm+ and SSm- MDR-TB patients diagnosed with different diagnostic algorithms. MDR-TB patients started treatment earlier if diagnosed with LPA in both SSm+ and SSm- groups.

The results of the analysis of time to treatment comparing the culture-based versus LPA-based algorithm are shown in Tables 2 and 3 and Fig 3. A reduction in median time from first care seeking visit to initiation of MDR-TB treatment was observed in both SSm+ and SSm- groups of patients diagnosed with LPA.

**Table 2:** Different time components from first care seeking visit to MDR-TB treatment initiation in SSm+ patients.

	Culture-based algorithm		LPA-based algorithm	p-value
	LJ (N = 38)	BacTAlert (N = 58)	LPA (N = 72)	
Median time from first care seeking visit to first microscopy (days, range) IQR	0 (0–350) IQR: 0–3.5	7 (0–576) IQR: 1.3–17.5	5 (0–172) IQR: 0–20	LJ vs. LPA: p = 0.001 BacTAlert vs. LPA: p = 0.484
Median time from first microscopy to sputum used for DST or LPA	0 (0–350)—IQR: 0–4	7 (0–576)—IQR: 1.8–16.5	5 (0–189) IQR: 1–20	LJ vs. LPA: p<0.001 BacTAlert vs. LPA: p = 0.779
Laboratory turn-around time	65 (43–100) IQR: 60–82	22 (11–49) IQR: 20–28	6 (2–94) IQR: 5–9	LJ vs. LPA: p<0.001BacTAlert vs. LPA: p<0.001
Median time from DST or LPA result to treatment (days, range)	13 (1–1238) IQR: 7–32.7	32 (8–969) IQR: 17–51	8.5 (1–416) IQR: 6–12	LJ vs. LPA: p = 0.003 BacTAlert vs. LPA: p<0.001
Overall median time from 1^st^ visit to treatment	90 (63–1321) IQR: 76.3–117.3	74 (31–990) IQR: 55–99.8	24 (6–511) IQR: 19–51	LJ vs. LPA: p<0.001 BacTAlert vs. LPA: p<0.001

**Table 3:** Different time components from first care seeking visit to MDR-TB treatment initiation in SSm- patients.

	Culture-based algorithm	LPA-based algorithm		p-value
	LJ (N = 67)	MGIT + LPA (n = 48)	LJ+LPA (n = 12)	
Median time from first care seeking visit to first microscopy (days, range) IQR	1 (0–770) IQR: 0–63.5	7.5 (0–209) IQR: 1.8–29	25 (6–168) IQR: 8.8–45	LJ vs. MGIT+LPA: p = 0.105 LJ vs. LJ+LPA: p = 0.020
Median time from first microscopy to sputum used for DST or LPA	2 (0–770) IQR: 0–74.8	7.5 (0–256) IQR: 1–29	25 (6–168) IQR: 8.8–45	LJ vs. MGIT+LPA: p = 0.322 LJ vs. LJ+LPA: p = 0.025
Laboratory turn-around time	74 (22–125) IQR: 64.3–84	28.5 (9–85) IQR: 23–35.3	64 (22–86) IQR: 54–72.3	LJ vs. MGIT+LPA: p<0.001 LJ vs. LJ+LPA: p = 0.020
Median time from DST or LPA result to treatment (days, range)	28 (6–262) IQR: 15–47.5	15 (5–493) IQR: 10–24.8	17 (13–42) IQR: 17–29.5	LJ vs. MGIT+LPA: p<0.001 LJ vs. LJ+LPA: p = 0.716
Overall median time from 1^st^ visit to treatment	140 (29–858) IQR: 99.5–216.5	62 (24–579) IQR: 50.3–84	113 (67–253) IQR: 88.5–131.8	LJ vs. MGIT+LPA: p<0.001 LJ vs. LJ+LPA: p = 0.037

The median time from the first care-seeking visit to treatment initiation was 24 (IQR 19–51) days for 72 SSm+ patients diagnosed by LPA (LPA-based algorithm), compared to 74 (IQR 55–99) days for 58 patients diagnosed by BacTAlert and 90 (IQR 76–117) days for 38 patients diagnosed by LJ (culture-based algorithm). The median reduction in time to treatment initiation by using LPA was 50 (BacTAlert, p<0.001) and 66 days (LJ, p<0.001).

For SSm- patients, the median time to treatment was 62 (IQR 50–84) days for 48 patients diagnosed by the MGIT+LPA, 113 (IQR 88–131) days for 12 patients diagnosed by LJ+LPA and 140 (IQR 99–216) days for 67 patients diagnosed by LJ. The median reduction of time of the treatment initiation was 78 (MGIT+LPA, p<0.001) and 27 days (LJ+LPA, p = 0.037).

Laboratory turn-around time of MDR-TB confirmation was the main component of overall time reduction and was reduced to 6 (IQR 5–9) days (LPA), compared to 22 (IQR 20–28) (BacTAlert) and 65 (IQR 60–82) (LJ) days in SSm+ group in culture-based-based algorithm (Table 2). In SSm- group laboratory turnaround time was 28.5 days (IQR 23–35) for LPA following MGIT, compared to 74 (IQR 64–84) days for LJ (Table 3).

### Impact of the introduction of LPA on smear and culture conversion

Comparative data on sputum smear and culture conversion at 2 and 6 months in patients diagnosed under the culture-based versus the LPA-based diagnostic algorithm are presented in Table 4. The implementation of LPA did not affect sputum smear conversion in MDR-TB patients compared to those diagnosed using either LJ or BacTAlert. Similarly, culture conversion rates at 2 and 6 months of treatment did not change between those groups. There was no difference in culture conversion in SSm- groups in LPA-based and culture-based algorithm at 2 and 6 months.

**Table 4:** Sputum and culture conversion rates at 2 and 6 months of treatment (*p*-values calculated for difference between proportions).

Diagnostic test	Smear conversion rate after 2 months of treatment	Culture conversion rate after 2 months of treatment	Culture conversion rate after 6 months of treatment
**Smear positive patients**			
LJ (n = 38)	42.1% (p = 0.078)	39.5% (p = 0.839)	68.4% p = (0.969)
BacTAlert (n = 58)	51.7% (p = 0.361)	36.2% (p = 0.879)	70.7% p = (0.746)
LPA* (n = 72)	54.7%	37.5%	68.1%
**Smear negative patients**			
LJ (n = 67)	-	65.7% (p = 0.702)	73.1% (p = 0.429)
LJ+LPA (n = 12)	-	66.7% (p = 0.943)	100%(p = 0.65)
MGIT +LPA* (n = 48)	-	70.8%	81.3%

*reference group

### Impact of introduction of LPA on treatment outcomes

Treatment outcomes at 20 months are presented in Table 5. Overall treatment outcomes were better in LPA-based algorithm compared to culture-based algorithm (p = 0.003). The implementation of LPA was associated with an increase in treatment success rates among MDR patients: 65.2% of patients diagnosed by LPA versus 44.8% patients diagnosed by LJ or BacTAlert. Accordingly there is a decrease in the number of patients who were lost to follow-up or died during treatment. Treatment failure and amplification of XDR-TB did not change significantly among the different patient groups.

**Table 5:** Treatment outcomes after 20 months of MDR-TB treatment comparing patients diagnosed based on different diagnostics in the LPA-based and culture-based algorithms (p = 0.003, Fisher’s exact test on the 6-by-2 table to evaluate whether the distribution in in treatment outcomes differed between culture-based and LPA-based algorithms. Treatment success was more common with the LPA-based algorithm, while lost to follow-up and all-cause mortality were more common with the culture-based algorithm).

	Treatment Success(cured and treatment completed combined)	Lost to follow up	All-cause mortality	Treatment failure	XDR (treatment failure)	Transfer out	Total
	Total number (%)	Total number (%)	Total number (%)	Total number (%)	Total number (%)	Total number (%)	
**culture-based algorithm cohort (all)**	**73(44.8)**	**53(32.5)**	**26(15.9)**	**5(3.1)**	**5(3.1)**	**1(0.6)**	**163**
SSm+ BacTAlert	19(32.8)	21(36.2)	10(17.2)	4(6.9)	4(6.9)	0(0)	58
SSm+ LJ	16(42.1)	13(34.2)	7(18.5)	1(2.6)	1(2.6)	0(0)	38
SSm- LJ	38(56.7)	19(28.4)	9(13.4)	0(0)	0(0)	1 (1.5)	67
**LPA-based algorithm cohort (all)**	**86 (65.2)**	**24 (18.1)**	**10 (7.6)**	**7 (5.3)**	**4 (3.0)**	**1 (0.8)**	**132**
SSm+	43 (59.7)	14 (19.4)	7 (9.7)	4 (5.6)	4 (5.6)	0 (0)	72
SSm- MGIT+LPA	34 (70.8)	8 (16.8)	3 (6.2)	3 (6.2)	0 (0)	0 (0)	48
SSm- LJ+LPA	9 (75.0)	2 (16.7)	0 (0)	0 (0)	0 (0)	1 (8.3)	12

## Discussion

Multiple recent studies from different settings, including studies in high MDR-TB burden countries, proved LPA to be an accurate, rapid tool for diagnosing MDR-TB that subsequently resulted in WHO policy recommendations [6,7,17]. Implementation of LPA in MDR-TB diagnostic algorithms was suggested to result in higher accuracy as well as shorter time to MDR-TB diagnosis [6,7,17]. However, there is little information on the impact of LPA on MDR-TB treatment initiation, improvement of treatment outcomes, as well as general improvement of TB transmission rates.

In our study, we demonstrated the positive impact of LPA implementation on time to MDR-TB treatment initiation and treatment outcomes in the high MDR-TB burden Arkhangelsk region of Russia, where LPA was introduced in 2009, replacing the culture-based algorithm [18].

The overall implementation of LPA led to earlier treatment initiation in both SSm+ and SSm- patients. Studies performed in South Africa [16,19], including another of the PROVE IT studies [16], and India [20] suggested similar results, although the overall time needed for treatment initiation was larger in those studies. This might be explained mainly by different approach in patients’ management in Arkhangelsk region, where all TB patients are considered to be presumptive MDR-TB cases and are tested initially for DST.

Main reduction in time from first care seeking visit to initiation of MDR-TB treatment was observed in laboratory turn-around time. In our study, laboratory procedures constituted approximately 25% of time (median 6 of total 24 days) needed for MDR-TB treatment commencement for LPA in SSm+ and 46% (median 28.5 of total 62 days) in SSm- groups and it is clear that time from first care-seeking visit to a registered MDR-TB case initiated on treatment is not only dependent on a tests’ laboratory turnaround time but impacted by different steps during patient’s diagnostic pathway [21].

Time from first care seeking visit to microscopy remained the same in both culture-based and LPA-based algorithms and seems to be unaffected by introduction of the new rapid tests in both SSm+ and SSm—groups. The overall time period between first-care seeking visit and first sputum smear microscopy to diagnose TB is relatively short (1 week) and is not creating large delays. At the same time, duration of this period varies greatly and 4 patients in this study entered the health care system with symptoms more than 1 year before receiving their first sputum microscopy while others received it within ≤ 1 day for group of patients that were evaluated with LJ. This might partly be explained by difficulties with establishing time when a person might be considered as TB suspect.

It is not surprising that time from first sample for smear microscopy to submission of sputum for LPA was the same (up to 7 days) compared to the period when sample was submitted to conventional DST, suggesting that the procedures and pathways prior to laboratory analysis are the same and different laboratory methods do not directly affect those pathways. At the same time, the combined time period from first care seeking visit to submission of sputum for DST or LPA can contribute to more than 2 weeks delay of treatment initiation.

Also since culture might still be needed prior to LPA, for example in SSm- patients, it is important to use rapid culture methods such as those liquid media—Bactec MGIT and BacTAlert in our study rather than solid media such as LJ.

Despite a considerable decrease in the turn-around time of MDR-TB laboratory diagnostics due to LPA implementation, further reduction could be expected with introduction of Cartridge Based Nucleic Acid Amplification testing such as Xpert MTB/RIF, especially for SSm- patients where culture might still be needed. At the same time, the PROVE IT study performed in South Africa suggested that reduction of laboratory turn-around time due to Xpert MTB/RIF to one day is still followed by delays in treatment commencement [16]. In our study few patients initiated treatment after more than 11 weeks in SSm+ group and more than 17 weeks in SSm- group following the first care-seeking visit in culture-based and LPA-based algorithm accordingly. Similar data was shown by Narasimooloo et al. [22]. In some cases, this might be explained by patients’ initial refusal of treatment after being diagnosed with MDR-TB and subsequent return to the healthcare as their condition worsens.

The time from LPA result to treatment was 8.5 days in SSm+ and 15 days in SSm- and was reduced in LPA-algorithm compared to time from DST result to treatment in culture-based algorithm in both SSm+ (23 days reduction compared to BacTAlert) and SSm- (13 days reduction), showing the impact of the change in the laboratory diagnostic method on the whole diagnostic algorithm, especially decision to treat. Although this time period was shorter compared to South African (14 days in SSm+ and 21 days in SSm-) and Indian (20 days in both in SSm+ and SSm-) studies [16, 19, 20] it might still be considered as a delay and certain organizational improvements might be warranted [19, 21]. For example, in the Arkhangelsk region medical committees for MDR registration could be held more frequently, e.g. daily instead of weekly. Similarly, the electronic reporting system of the laboratory results could be implemented in a way that ensures clinicians receive all results, including MDR confirmation, without the delays and losses observed in paper-based reporting systems.

The results also showed that SSm+ patients begin MDR treatment earlier than SSm- because they are hospitalized to ACAD earlier than SSm-. The SSm- patients initiate treatment for TB in the ambulatory units before they are diagnosed with MDR-TB and are then hospitalized to ACAD for a change of treatment. Therefore, the SSm- patients experience a delay in initiation of MDR treatment. Additionally it is possible that using LJ instead of BacTAlert caused a delay in MDR-TB diagnostic in SSm- group of patients.

Despite a shortened time to treatment, there was no significant difference in sputum and culture conversion rates at 2 and 6 months of treatment, although the numbers were small. We suggest that the average time reduction for diagnosis of MDR-TB using LPA has an impact on treatment outcomes, but has less of an effect on smear and culture conversion rates in the early phases of treatment.

MDR-TB affects treatment outcomes, and treatment success rates are lower in MDR-TB patients compared to non-MDR patients [17]. According to the WHO, the proportion of MDR-TB patients in the 2007 cohorts ranged from below 40% to almost 80% in different countries [23]. Recent WHO data showed that in 2010 the number of MDR-TB patients who successfully completed treatment was 48% with higher treatment success rates among patients in the Eastern Mediterranean Region (56%) and the Americas Region (54%) [2]. In studies from Tomsk, Orel and Vladimir regions of the Russian Federation, successful treatment of MDR-TB patients reached over 60% [24, 25]. In our study 44.8% of patients in the historical cohort had successful treatment outcome, which correlates with average rates in different settings [26, 27], but is lower compared to the outcomes in other regions of Russian Federation. At the same time, patients diagnosed under the LPA-based algorithm had higher successful treatment rates (65.2%). A similar successful treatment rate was seen in other studies [28]. Lower lost to follow up and all-cause mortality rates were also registered in the patients diagnosed with the LPA-based algorithm.

The main limitation of our study was the use of a historical cohort for comparison. Although treatment regimens for MDR-TB patients remained unchanged for patients diagnosed by both the culture-based and LPA-based algorithms, other undetected factors, such as the epidemiology of TB in the region, might have biased the results. For example, TB specialists’ awareness of LPA-based algorithm to detect MDR-TB cases more quickly might encourage more active case finding as well as quicker decision making, thereby resulting in shorter time to treatment initiation without being directly affected by LPA. Additionally, lost to follow up rates were also lower in LPA-based algorithm and other factors might contribute to that, although no changes in program of MDR-TB management, such as social support, were introduced. On the other hand we suggest that switching to MDR-TB treatment after several weeks of non-MDR-TB treatment under the culture-based algorithm might discourage patients from continuing their treatment and result in default. Patients’ defaulting from MDR-TB treatment is an important issue for all TB programs. We were not able to perform a randomized-controlled study in our high MDR-TB burden setting because LPA was introduced in MDR-TB diagnostic algorithm for all TB patients in our region, as recommended by WHO. The introduction of LPA was the main change to MDR-TB management and is likely to have been the major contributor to the observed improvements.

## Conclusion

We have shown that introduction of the new LPA-based diagnostic algorithm is associated with earlier initiation of treatment as well as better treatment outcomes for patients with MDR-TB. SSm+ MDR-TB patients were those who benefited the most from the introduction of LPA, lesser improvements were observed in SSm- group. LPA leads to earlier initiation of treatment, yet the time for pre-laboratory, before submission for DST, is not affected by introduction of the rapid tests and impacts the timely initiation of MDR-TB treatment. But the results also suggest that pre- and post- laboratory patient and health system pathways and decision-making need to be further optimized to improve patient management and treatment initiation.
